# Rapid adsorptive removal of chromium from wastewater using walnut-derived biosorbents

**DOI:** 10.1038/s41598-023-33843-3

**Published:** 2023-04-26

**Authors:** Rajni Garg, Rishav Garg, Mika Sillanpää, Alimuddin, Mohammad Amir Khan, Nabisab Mujawar Mubarak, Yie Hua Tan

**Affiliations:** 1 Department of Applied Sciences, Galgotias College of Engineering and Technology, Greater Noida, Uttar Pradesh 201310 India; 2 Department of Civil Engineering, Galgotias College of Engineering and Technology, Greater Noida, Uttar Pradesh 201310 India; 3 Department of Chemistry, College of Science, King Saud University, Riyadh, 11451 Saudi Arabia; 4 Department of Chemical Engineering, School of Mining, Metallurgy and Chemical Engineering, University of Johannesburg, P. O. Box 17011, Doornfontein, 2028 South Africa; 5 Zhejiang Rongsheng Environmental Protection Paper Co. LTD, NO.588 East Zhennan Road, Pinghu Economic Development Zone, Pinghu, Zhejiang 314213 People’s Republic of China; 6 Physical Sciences Section, School of Sciences, Maulana Azad National Urdu University, Hyderabad, 500032 Telangana India; 7 Petroleum and Chemical Engineering, Faculty of Engineering, Universiti Teknologi Brunei, Bandar Seri Begawan, BE1410 Brunei Darussalam; 8 Department of Chemical and Energy Engineering, Faculty of Engineering and Science, Curtin University Malaysia, CDT 250, 98009 Miri, Sarawak Malaysia

**Keywords:** Environmental sciences, Environmental social sciences, Chemistry, Energy science and technology, Engineering

## Abstract

Contamination of water resources by industrial effluents containing heavy metal ions and management of solid waste from agricultural and food industries is a serious issue. This study presents the valorization of waste walnut shells as an effective and environment-friendly biosorbent for sequestrating Cr(VI) from aqueous media. The native walnut shell powder (NWP) was chemically modified with alkali (AWP) and citric acid (CWP) to obtain modified biosorbents with abundant availability of pores as active centers, as confirmed by BET analysis. During batch adsorption studies, the process parameters for Cr(VI) adsorption were optimized at pH 2.0. The adsorption data were fitted to isotherm and kinetic models to compute various adsorption parameters. The adsorption pattern of Cr(VI) was well explained by the Langmuir model suggesting the adsorbate monolayer formation on the surface of the biosorbents. The maximum adsorption capacity, *q*_*m,*_ for Cr(VI) was achieved for CWP (75.26 mg/g), followed by AWP (69.56 mg/g) and NWP (64.82 mg/g). Treatment with sodium hydroxide and citric acid improved the adsorption efficiency of the biosorbent by 4.5 and 8.2%, respectively. The endothermic and spontaneous adsorption was observed to trail the pseudo-second-order kinetics under optimized process parameters. Thus, the chemically modified walnut shell powder can be an eco-friendly adsorbent for Cr(VI) from aqueous solutions.

## Introduction

The water contamination by effluents generated by various process industries containing non-degradable and persistent heavy metals is a global concern due to its severe negative impact on the environment^1^. Heavy metals, such as As, Pb, Cr, Hg and Ni, are non-biodegradable, toxic and persist in the environment due to their bioaccumulation tendency causing severe health issues to live organisms upon entering the food chain^2^. Cr, predominantly as Cr(VI), is among the most prevalent water contaminant generated by the mining, metal finishing, textile, electroplating, and leather industries^3–5^. As per WHO regulations, the safe and permissible Cr(VI) concentration in drinking water and industrial wastewater is 0.05 mg/L and 0.5 mg/L, respectively^5^. Cr(VI) is highly soluble in water and can enter the human body through dermal and oral exposure. It is extremely toxic and is carcinogenic in long-term exposure and must be removed from the effluents before disposal^6^.

The conventional techniques reported for the sequestration of Cr(VI) include solvent extraction^7^, filtration^8^, reduction^9^, precipitation^10^, and ion exchange^11^. Still, these methods have limited applicability due to the requirement of energy and cost-intensive instruments, hazardous chemical reagents, and, in some cases, their application results in secondary pollution^12^. The requirement of monitoring and disposal of generated solids and sludge requires additional labor, and the process may result in incomplete treatment limiting its suitability for large volumes^12^. Biosorption has been considered a simple, easy-to-operate, cost-effective and eco-friendly technique with bio-degradable and inexpensive materials^13^. These materials include animal wastes (waste egg shells and bones)^14^, microbial biomass (algae and bacteria)^5^, and agricultural waste (bark, leaves, fruit peels, seeds, husk, shells, straws, etc.)^15^. The efficiency of biosorbents can be enhanced by chemical treatment with acids and bases, resulting in modification of the functionalities present at the biosorbent surface and enhancing active sites^16^.

Agricultural wastes have been recognized as low-cost, renewable, biodegradable and eco-friendly biosorbents with significant adsorption capacity for contaminants such as heavy metal ions, pharmaceutical products, dyes and aromatic compounds^17^. Agricultural waste as a biosorbent also provides a sustainable solution for efficient management and utilizing this ever-growing waste generated by agricultural operations, domestic food preparation and industrial food processing^18^. Agricultural waste is a rich source of lignocellulosic material, viz. lignin, cellulose, hemicellulose, pectin, proteins, flavonoids, terpenoids and other secondary metabolites having polyhydroxy, carboxy, amine, and aldehydic, functionalities with high affinity for metal ions^15^. Agricultural wastes, including rice husk^19^, wheat bran^20^, palm kernel shell^21^, apricot seed^16^, groundnut hull^22^, leaves^1^, peels of fruits and vegetables^23^, vegetable waste^24^, and bagasse^25^ as a dried powder or ash have been reported as effective adsorbents for persistent organic compounds and heavy metal ions due to their greater adsorption efficacy as well as the easiness of separation and regeneration^26,27^.

Walnut (*Juglans regia*) is consumed as a highly nutritious nut worldwide. Its shell is discarded as worthless agricultural waste after the removal of its kernel and is a rich source of lignocellulose comprising lignin (36.90%), hemicellulose (36.06%), and cellulose (17.74%)^28^. Literature reports that walnut shell is an inexpensive and effective biosorbent for sequestering contaminants such as heavy metal ions^30^, dyes^29^, and other organic compounds^31^ from wastewater. In the case of lignocellulosic materials, cellulose and hemicelluloses are in a compact lattice form, limiting the availability of free hydroxyl groups for binding with heavy metal ions. Hence, their adsorption capacity is quite low^16^. The efficiency of such biosorbents can be increased after chemical modification with sodium hydroxide (base) and citric acid (acid). Sodium hydroxide disintegrates this lattice, making hydroxyl groups easily accessible on the biosorbent surface^28^. Citric acid is considered more beneficial in increasing the adsorption efficiency of lignocellulosic materials. It has tricarboxylic groups, one of which reacts with the hydroxyl group of the cellulose present in the walnut. In contrast, two groups remain available for reacting with heavy metal ions, thereby increasing the adsorption capacity of the adsorbent^30^.

Literature reports the use of walnut shells as biochar or activated carbon to sequestrate Cr(VI); however, reports on walnut shell powder in native form and chemically modified form are scanty^31^. This paper compares the effect of chemical modification of native walnut powder by sodium hydroxide and citric acid on the adsorption capacities of the native and chemically treated walnut shell powder to sequestrate Cr(VI) from aqueous solutions. The functional characteristics of these inexpensive biosorbents and adsorption behavior were studied with process parameters optimization. The underlying mechanism has been explored through kinetics and thermodynamic analysis.

## Materials and methods

### Materials

The chemicals in their analytical grade were purchased from Sigma-Aldrich (Delhi) and utilized in the study. The walnut shells were obtained as a waste product from a local vendor. The shells were washed in deionized water to remove dirt or debris, laid out in the sun to dry completely and then milled into a powder for further use in three separate sets.

### Methods

The powdered shell was sieved in the first set to obtain native walnut shell powder (NWP). In the second set, 20 g of NWP was treated with 2 M sodium hydroxide solution at 353 K with constant stirring for 2 h. using a magnetic stirrer. The solution was filtered, and the filtrate was dried in a hot air oven at 383 K after rinsing with de-ionized water. The dried powder was stored for later usage as alkali-treated walnut shell powder (AWP). In the third set, 20 g of NWP was treated with 2 M citric acid solution instead of sodium hydroxide while following the same procedure as in the second step to obtain citric acid-treated walnut shell powder (CWP).

The examination of the surface morphology of the three biosorbents (NWP, AWP, and CWP) was executed using a scanning electron microscope (SEM, Model ZEISS EVO 50) and XRD (Model PANalytical X'Pert Pro) operating with a continuous speed of 0.045° per min. At 45 kV using Cu–Kα radiation (K = 1.5406 A°). The associated functional groups were identified using a Fourier transform infrared spectroscope (FTIR, Model NICOLET-IS-50). The surface area and pore volumes of the three biosorbents were measured on Brunauer–Emmett–Teller (BET) surface area analyzer (Model BELSORP-maxII). At the same time, the zeta potential was evaluated by a zeta potential analyzer (Malvern Zetasizer, Model Nano ZS). the pH of the prepared solutions was monitored through a pH meter (Model 1010 Labtronics). The residual concentration of Cr(VI) was measured using an atomic absorption spectrophotometer (PerkinElmer AAS, Model PinAAcle 900 T).

**Figure 1:**
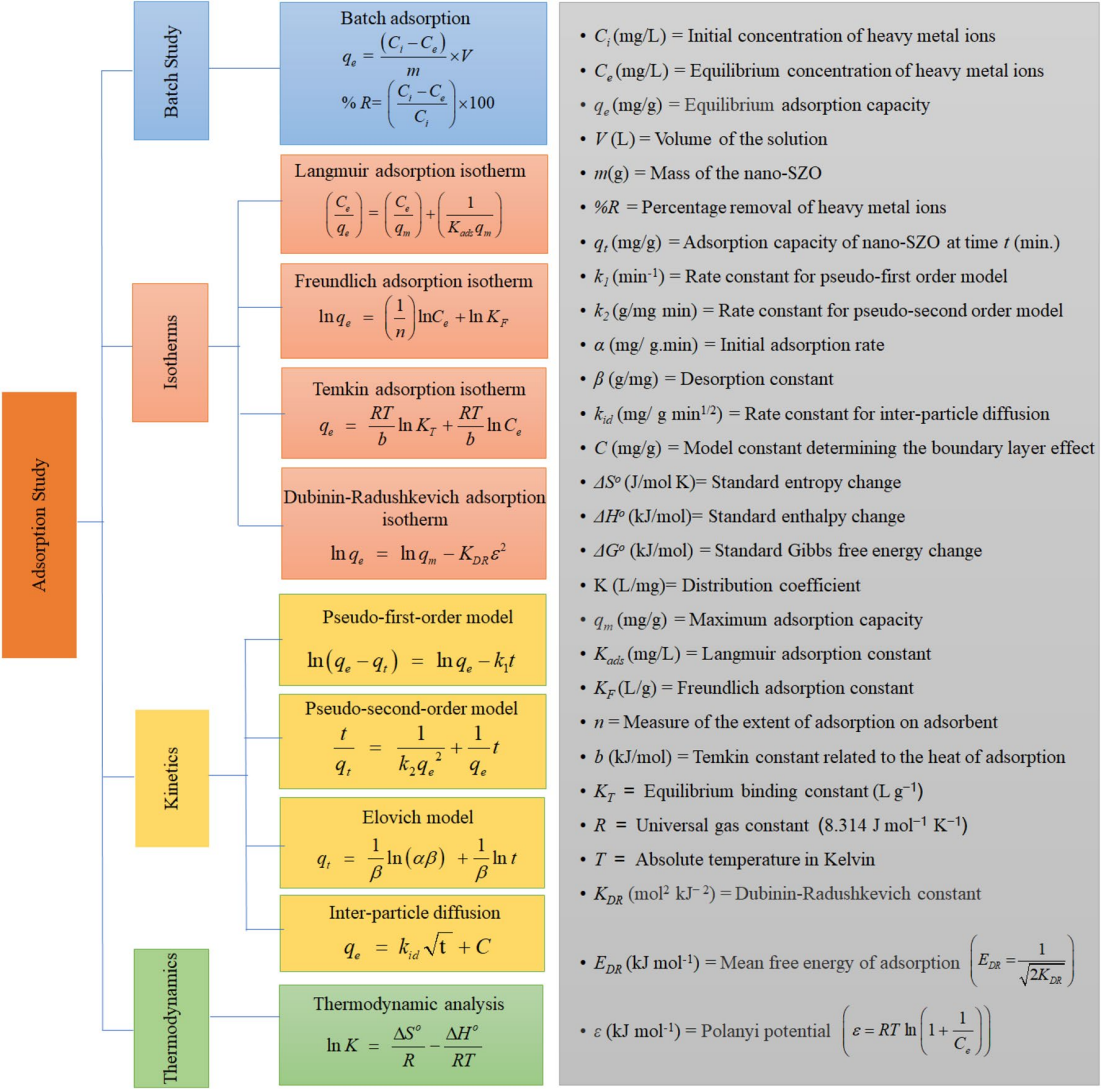
Program for adsorption studies.

Synthetic media containing 20 mg/L Cr(VI) were prepared and diluted using de-ionized water to obtain the necessary concentration. Experiments were carried out in triplicates using batch procedures, which involved adding a known amount of biosorbent (0.2–1.2 g/L), to 100 mL of Cr(VI) solution, with agitation at 298 K for 120 min. pH was adjusted to the desired range (1.0–8.0) using aliquots of dilute hydrochloric acid (HCl) or sodium hydroxide (NaOH), and then the mixture was allowed to equilibrate. Analyses were conducted at a temperature range of 288–328 K. After filtering the sludge, the concentration of the residual ions was measured. Sludge-exhausted biosorbent was regenerated using 0.1 M HCl. The adsorption data were analyzed through the program for isotherm and kinetic analysis models, as illustrated in Fig. 1. The results were analyzed through linear and non-linear regression analysis to identify the appropriate isotherm and kinetic models based on maximum values of the regression coefficient (R^2^) along with minimum values of chi-square (χ^2^) and Marquardt’s percent standard deviation (MPSD)^32^.

## Results and discussion

### Characterization of the biosorbents

**Figure 2:**
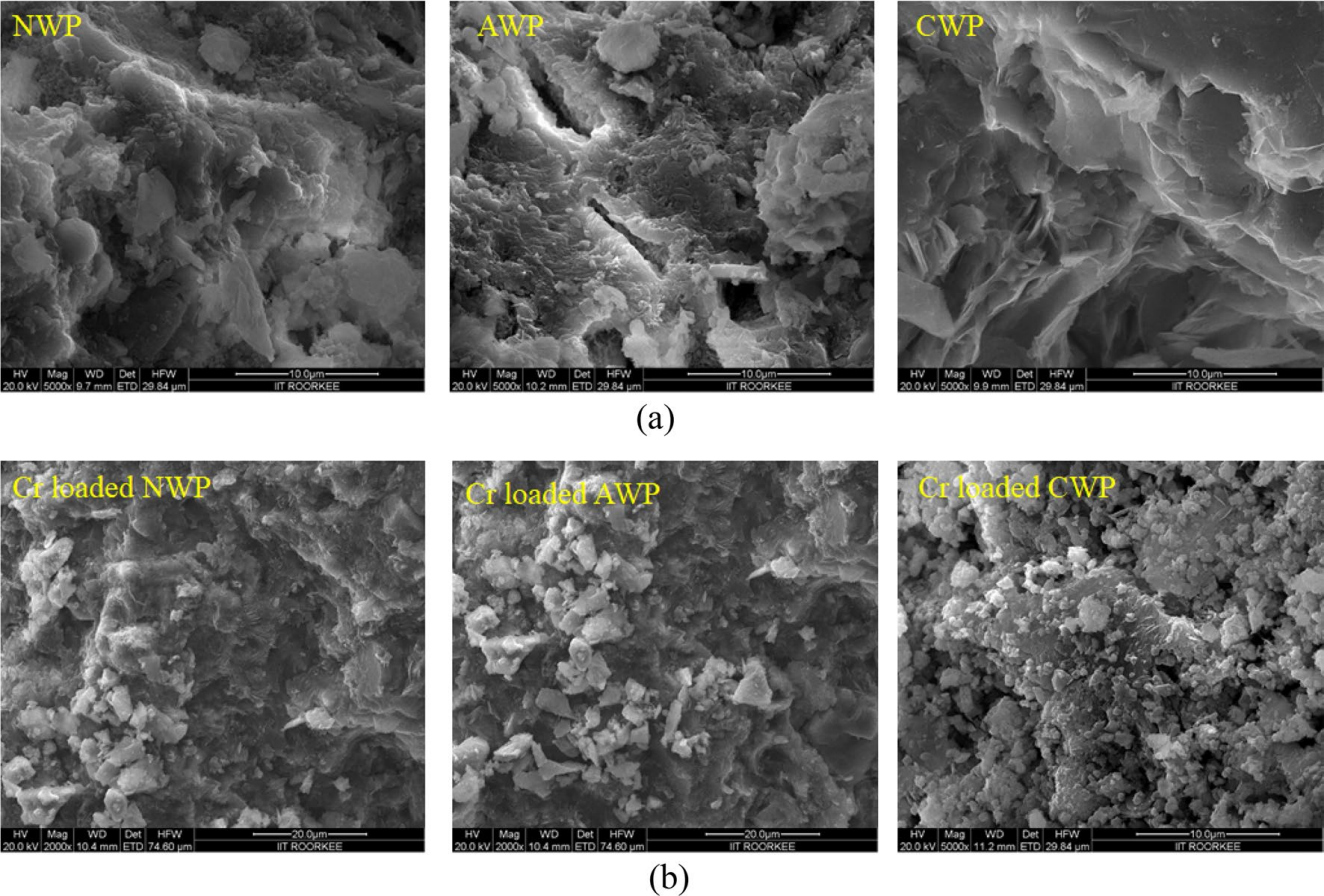
(**a**) Micrographs of native biosorbents (**b**) micrographs of Cr-loaded biosorbents.

The micrographs of the NWP, AWP, and CWP are shown in Fig. 2a, illustrating the surface modifications in the biosorbents with treatment. The heterogeneous porous and rigid structure of NWP transformed into a ruptured structure with alkali treatment (AWP) and exhibited more homogeneity with enlarged pores after treatment with citric acid (CWP). The micrographs of the three biosorbents showed clogged pores after the biosorption of Cr(VI) ions, as evident in Fig. 2b.

**Figure 3:**
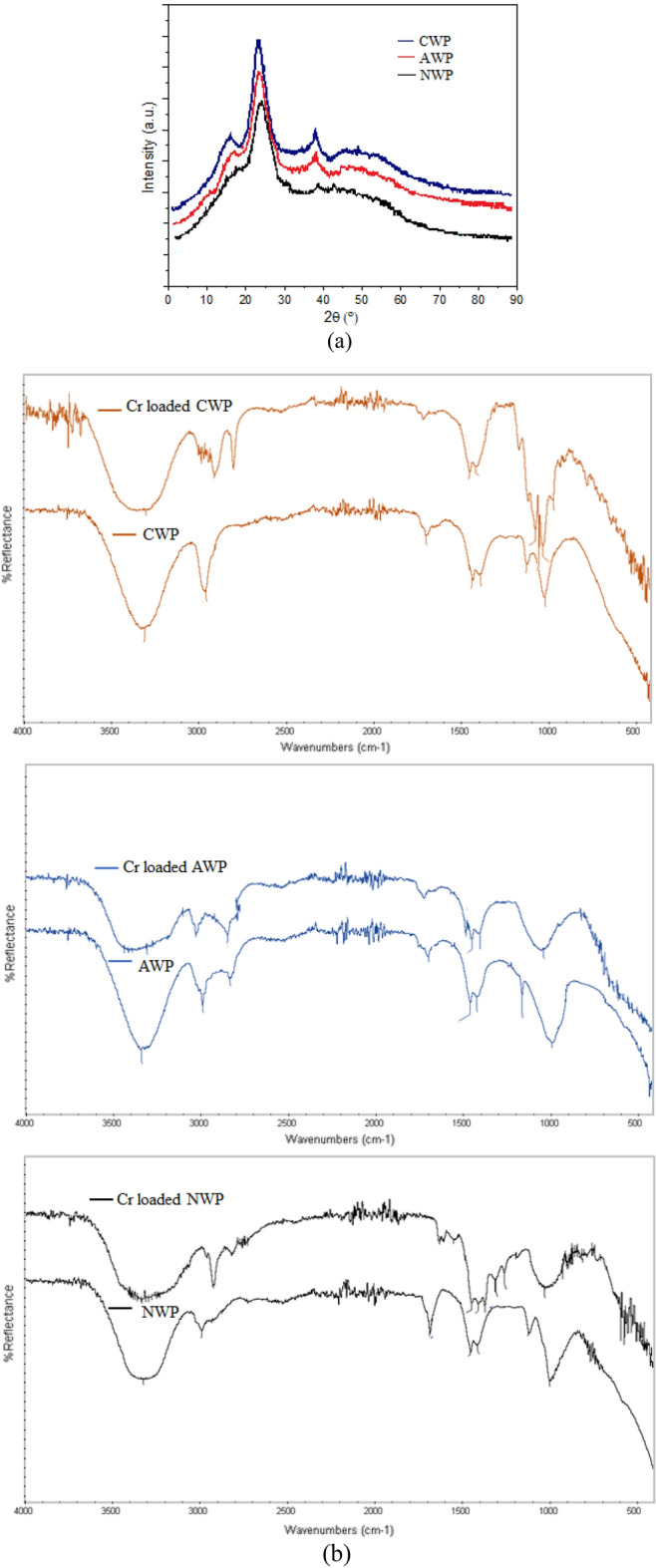
(**a**) XRD of native biosorbents (**b**) FTIR spectra of native and Cr-loaded biosorbents.

Figure 3a represents the XRD patterns for the three biosorbents with characteristic peaks for polysaccharides and cellulose^33^. A change in the intensity of the peaks was observed with chemical modification indicating a difference in the lattice. BET surface area for the three biosorbents was obtained as 423.86 m^2^/g (NWP), 567.31 m^2^/g (AWP), and 602.47 m^2^/g (CWP), with BJH pore volumes equal to 0.426 cm^3^/g (NWP), 0.575 cm^3^/g (AWP), and 0.641 cm^3^/g (CWP), indicating abundant availability of pores as active centers for higher adsorption of Cr(VI) onto nano-CWP. FTIR spectra of the three biosorbents (NWP, AWP and CWP) before and after sorption of Cr(VI) ions have been illustrated in Fig. 3b. In native NWP, AWP, and CWP, the biosorption peaks around 3260.53 cm^−1^, 3227.48 cm^−1^ and 3229.11 cm^−1^ reflected the presence of OH groups^5^. The peaks at 2976.12 cm^−1^ (NWP), 2918.76 cm^−1^ (AWP) and 2900.38 cm^−1^ (CWP) were attributed to C–H stretching modes^3^. The peaks around 1724.12 cm^−1^ (NWP), 1716.36 cm^−1^ (AWP) and 1603.57 cm^−1^ (CWP) corresponded to the C = O stretching, while the peaks at 1581.18 cm^−1^ (NWP), 1594.06 cm^−1^ (AWP) and 1606.47 cm^−1^ (CWP) were associated to N–H bending modes^5^. The peaks at 1298.12 cm^−1^ (NWP), 1270.40 cm^−1^ (AWP) and 1242.35 cm^−1^ (CWP) were attributed to stretching modes of the C–O group in lignocellulosic compounds^30^. The peaks at 1042.55 cm^−1^ (NWP), 1008.42 cm^−1^ (AWP) and 1052.33 cm^−1^ (CWP) were related to the bending modes of the C–N group^5^. The shifts in the characteristic peaks (Fig. 3b) after biosorption of Cr(VI) confirm the participation of various functionalities in metal binding^4^.

### Batch adsorption studies

**Figure 4:**
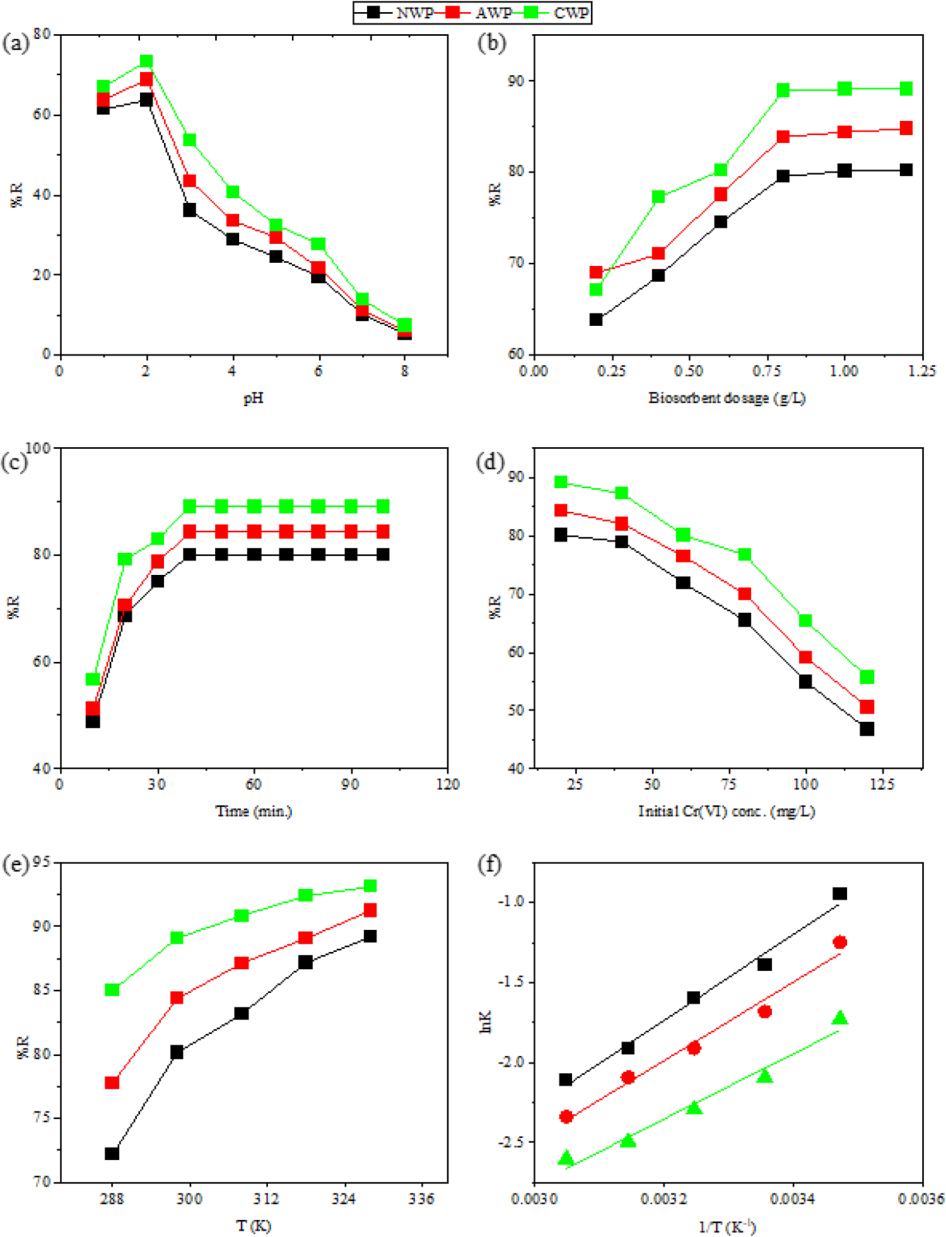
Effect of (**a**) pH (**b**) biosorbent dosage (**c**) contact time (**d**) initial concentration of Cr(VI) (**e**) temperature variation and (**f**) Van’t Hoff plots for adsorption of Cr(VI).

the pH of the solution significantly impacts the adsorption rate because the surface characteristics of the adsorbent change considerably with a change in pH. The solution pH was varied from 1.0 to 8.0 for an initial concentration of Cr(VI) as 20 mg/L at a constant biosorbent dose (0.2 g/L) with agitation at 200 rpm for 120 min at 298 K. Figure 4a illustrates an initial increase in biosorption of Cr(VI) by NWP, AWP and CWP with an increase in pH from 1.0 to 8.0 followed by a progressive decrease with maximum biosorption at pH 2.0. CWP has shown the maximum Cr(VI) removal of 73.40%, followed by AWP (68.98%) and NWP (63.78%). Cr(VI) exists in many forms viz. the neutral H_2_CrO_4_ at pH < 2.0, anionic HCrO_4_^−^ and Cr_2_O_7_^2−^ at 7.0 > pH < 2.0 while CrO_4_^2−^ as the predominant form at pH > 7.0^9^. The polyhydroxy, carboxy and amine functionalities in the biosorbents facilitate the adsorption by participation in metal ion binding^30^. The pH at a zero-point charge (pH_pzc_) for the biosorbents was determined as 4.9 (NWP), 5.4 (AWP) and 4.2 (CWP). The functionalities present at the biosorbent surface get protonated at lower pH (< pH_pzc_), resulting in the positively charged surface leading to an electrostatic attraction towards the negatively charged forms of Cr(VI) and increased biosorption^3^. Literature reports HCrO_4_^−^ as the most prevailing anionic Cr(VI) form in an aqueous medium at pH 2.0–4.0^34^. Thus, maximum biosorption at pH 2.0 confirms HCrO_4_^−^ as the predominant species and the optimum pH of 2.0 for further study. Deprotonation of the functionalities with increased pH (> pHpzc) and the possible competition between the anionic species and OH − ions in the solution decreases the biosorption capacity^21^.

The amount of adsorbent needed to remove a given substance affects its removal capacity. The biosorbent dose was varied (0.2–1.2 g/L) for an initial concentration of Cr(VI) as 20 mg/L at 298 K and optimized pH (2.0) for 120 min. (Fig. 4b). The biosorption increased with an increase in biosorbents dosage and a decrease in equilibrium time. The biosorption increased from 63.78% to 80.12% for NWP, 68.98% to 84.35% for AWP and 67.13% to 89.08% for CWP as the dose increased from 0.2 to 1.0 g/L, possibly with the accessibility of more active sites at higher biosorbent dose^18^. Significant removal of Cr(VI) was accomplished within 40 min. and no further substantial change in biosorption was observed, indicating the attainment of equilibrium. Thus, a dose of NWP, AWP and CWP was used at 1.0 g/L in later studies. The adsorption efficiency of the biosorbents exhibited no significant change with further increase in dosage, possibly due to the overlapping of the active sites with over-crowding of the biosorbent particles^12^. Vu et al. 2019 also obtained a similar trend in the adsorption of Cr(VI) onto biosorbent derived from snail shell^3^.

A biosorbent dosage of 1.0 g/L was used for studying the effect of contact duration at the optimized pH and 298 K. The adsorptive uptake of Cr(VI) from the solutions (20–120 mg/L) onto NWP, AWP and CWP was found to be dependent on the initial concentration of Cr(VI) as well as contact time. Figure 4c shows the impact of contact time on biosorption under optimized process parameters, while Fig. 4d shows the outcome of varying the initial concentration of Cr(VI) on biosorption. An initial increase in biosorption attained a maximum value at about 40 min. (attainment of equilibrium) with no significant change after that. Similar results have been reported by Bansal et al. 2022 for time-dependent adsorption of Cr(VI) in their study^18^. It may be attributed to the exhaustion of the active sites for the constant dose of the biosorbents at equilibrium. Thus, 40 min. It was considered the optimum time for biosorption under the studied parameters. The percentage removal decreased as the concentration of Cr(VI) in the solution was increased from 20 mg/L to 120 mg/L. The decrease in percentage removal can be linked to the lower ratio of the active sites and the metal ions, leading to the exhaustion of the active sites of the biosorbents. Further, the repulsive forces between the adsorbate and bulk phase also decreased the uptake of the metal ions^16^.

### Desorption and regeneration analysis

The biosorbents exhibited significant ease of regeneration and reusability in the successive adsorption and desorption cycles. An adsorbent’s reusability and desorption efficiency depends upon its binding efficiency with the adsorbates^13^. Maximum desorption efficiency was exhibited by NWP, followed by AWP and CWP, indicating greater binding of Cr(VI) onto the CWP surface. The biosorbents were found effective up to ten cycles of successive adsorption and desorption studies with a slight reduction in percentage removal, indicating their significant potential as bioadsorbents.

### Thermodynamic analysis

The variation of percentage removal of Cr(VI) (20 mg/L) by NWP, AWP and CWP at optimized process parameters with a variation of temperature (288–328 K) is shown in Fig. 4e. A gradual increase in the removal percentage from 72.15 to 89.19% (for NWP), 77.75 to 91.22% (for AWP) and 84.96 to 93.11% (for CWP) was observed on increasing the temperature. The maximum percent removal was found in CWP, followed by AWP and NWP. The results reflect the endothermic nature of the sorption due to the increased transport of metal ions with the increasing temperature that also increases the number of active sites with cleavage of bonds at the surface of the adsorbent^35^. The linear plot of lnK vs. 1/T (Fig. 4f) was used to compute the thermodynamic parameters (Supplementary information as shown in Table S1). The negative values of ΔH^o^ validate the endothermic nature of the adsorption, while the increase of randomness at the surface of biosorbents is indicated by the positive values of ΔS^o^^36^. The negative values of Δ*G*˚ confirm that the adsorption of Cr(VI) onto NWP, AWP and CWP is spontaneous. Many researchers have reported automatic Cr(VI) adsorption onto biosorbents such as lignocellulosic nanocomposites^37^, apricot shell^30^, and peanut shell^15^.

### Isotherm studies

**Figure 5:**
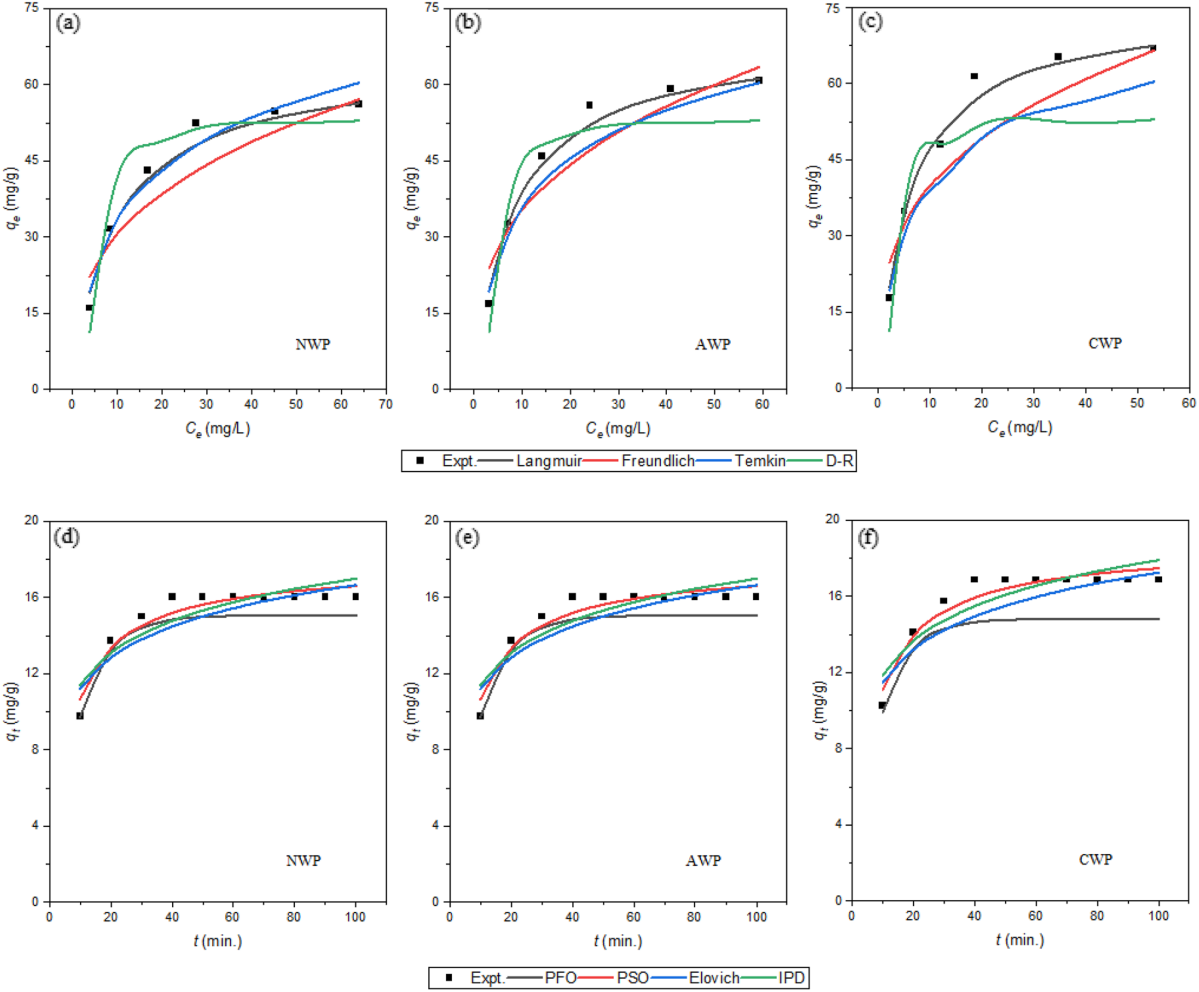
Comparison of experimental and calculated values of *q*_*e*_ and *q*_*t*_ after non-linear regression analysis of isotherms and kinetic models, respectively, for adsorption of Cr(VI) onto biosorbents.

Figure 5a–c shows the fitted plots of the four isotherm models for the adsorption of Cr(VI) (20–120 mg/L) onto NWP, AWP, and CWP (1.0 g/L) at optimized pH at 298 K. Table 1 shows the values of the obtained isotherm parameters after non-linear regression analysis. The data fitted best with the linearized expression of Langmuir isotherm, with the highest values of R^2^ and lowest values of χ2 and MPSD, indicating the model's efficacy in describing the equilibrium data with monolayer coverage of Cr(VI) on the homogeneous surface of the biosorbents^38^. The maximal adsorption capacity, q_m_, was found to be highest for CWP (75.26 mg/g), followed by AWP (69.56 mg/g) and NWP (64.82 mg/g). A similar trend was observed for the adsorption parameter *K*_*ads*_, in the order of CWP (0.1633 L/mg), AWP (0.1235 mg/g), and NWP (0.1044 mg/g). This parameter signifies the affinity of the biosorbent for the adsorbed metal ions. The trend confirms the positive impact of treatment with citric acid is consistent with results reported in the literature^30^.

**Table 1:** Isotherms constants for the adsorption of Cr(VI) on biosorbents.

Model	Parameters	NWP	AWP	CWP
Langmuir adsorption isotherm	K_ads_ (L/mg)	0.1044	0.1235	0.1633
	q_m_ (mg/g)	64.82	69.56	75.26
	*R*^*2*^	0.9730	0.9826	0.9829
	χ^2^	0.0531	0.0005	0.0144
	MPSD	0.2826	1.8327	2.5945
Freundlich adsorption isotherm	K_F_ (mg/g)	13.93	16.38	19.46
	*n* (L/mg)	2.94	3.01	3.23
	*R*^*2*^	0.8416	0.8745	0.8328
	χ^2^	0.2662	0.0516	0.2070
	MPSD	6.6936	5.1037	10.1356
Temkin adsorption isotherm	K_T_ (L/mg)	0.93	1.15	1.60
	*b* (kJ/mol)	0.167	0.160	0.151
	*R*^*2*^	*0.9554*	0.9610	0.9601
	χ^2^	1.5582	1.4082	1.4969
	MPSD	7.9380	7.1343	10.6381
D–R adsorption isotherm	K_DR_ (× 10^−6^ mol^2^/J^2^)	5.0169	3.6198	2.1038
	q_m_ (mg/g)	53.29	56.97	62.32
	E_DR_ (kJ/mol)	0.36	0.36	0.36
	*R*^*2*^	0.9322	0.9085	0.8814
	χ^2^	2.9301	4.8144	6.9973
	MPSD	11.0163	15.2672	15.2791

The possibility of multilayer adsorption considering the heterogeneous surface of biosorbent was explored through a linear fit of the equilibrium adsorption data with the Freundlich adsorption isotherm. The model fitted well with the value of *R*^*2*^ (Table 1), lesser than that for the Langmuir model, reflecting the model’s non-suitability for the adsorption behavior. The value of the Freundlich constant, *K*_*F*_, indicative of the relative adsorption capacity, was found to be highest for CWP (19.46 mg/g) as compared to AWP (16.38 mg/g) and NWP (13.93 mg/g). The differential affinity of NWP, AWP and CWP for Cr(VI) was reflected by varying values of n, a measure of the extent of adsorption^1^. The corresponding values lying between 1 to 10 (Table 1) indicated favorable adsorption with chemical modification of the available sites.

The mean free energy of adsorption $E_{DR}$ was computed by fitting the adsorption data to the Dubinin-Radushkevich isotherm model. The values were less than 8 kJ/mol indicating physisorption on the surface of the biosorbents^37^. The value of *R*^2^ was lesser than that of the Langmuir model, while that of χ^2^ and MPSD were higher, showing the non-suitability of the model for explaining the adsorption process for the studied systems. The equilibrium adsorption data was also used to fit the Temkin adsorption isotherm model that considers adsorbent–adsorbate interactions (Fig. 4d). The values of Temkin constant, *b*, the measure for the heat of adsorption, were computed by fitting the adsorption data in the Temkin isotherm model. The corresponding values < 8 kJ/mol supported the physisorption of metal ions onto the three biosorbents^12^. However, this model was also not considered suitable for the current study due to comparatively low values of *R*^2^ and high values of χ2 and MPSD compared to the Langmuir model^39^.

The maximum adsorption capacities of the studied biosorbents have also been compared with that of other biosorbents reported in the literature and listed in Table 2. The comparative account shows the studied biosorbents' effectiveness as potential Cr(VI) sequestration agents.

**Table 2:** A comparative account of adsorption capacities of walnut shell derived biosorbents with other biosorbents.

Adsorbent	Adsorption capacity (mg/g)	Reference
Diethylenetriamine-modified walnut shell	50.1	^33^
Phosphoric acid-modified walnut shell	39.22	^40^
Modified corn stalk biochar	25.6	^41^
Eucalyptus bark biochar	10	^42^
Peanut shell	2.48	^15^
Walnut shell	41.53	^43^
Groundnut Shell activated – aluminum embedded carbon	13.458	^44^
Walnut shell derived biosorbents (NWP, AWP and CWP)	64.82–75.26	This work

### Kinetics studies

The adsorption behavior of Cr(VI) (20 mg/L) onto the three biosorbents (1.0 g/L) was explored for 120 min. at the optimized pH and 298 K, the data was applied to kinetic models followed by non-linear regression analysis to obtain the fitted parameters listed in Table 3. Figure 5d–f elucidates comparing the experimental and calculated qt values after the non-linear regression analysis. The analysis shows the better fit of biosorption data with pseudo-second-order kinetics with the highest values of *R*^2^ along with the lowest values of χ^2^ and MPSD. The good agreement between experimental and calculated qe values further supported pseudo-second-order kinetics. Several researchers have reported the adsorption process of Cr(VI) onto biosorbents to follow second-order kinetics^39,45^.

**Table 3:** Kinetics models parameters for adsorption of Cr(VI) on biosorbents.

Biosorbents		NWP	AWP	CWP
Experimental	Q_e_ (mg/g)	16.02	16.87	17.82
Pseudo-first-order model	k_1_ (/min)	0.1037	0.1107	0.1157
	Q_e_ (mg/g)	15.07	14.82	16.68
	*R*^*2*^	0.8809	0.8744	0.8753
	Χ^2^	0.0227	0.0595	0.0409
	MPSD	37.240	74.7184	37.3130
Pseudo-second-order model	k_2_ (g/mg.min)	0.0086	0.0078	0.0088
	Q_e_ (mg/g)	17.68	18.66	19.45
	*R*^*2*^	0.9932	0.9937	0.9183
	Χ^2^	0.0135	0.0032	0.0370
	MPSD	2.393	2.2098	2.0064
Elovich model	α (mg/g.min)	26.67	24.10	50.79
	β (g/mg)	0.4221	0.3981	0.4160
	*R*^*2*^	0.786	0.803	0.771
	Χ^2^	0.6350	0.7500	0.6495
	MPSD	4.568	9.6294	5.0500
Inter-particle diffusion model	k_id_ (mg/g min^1/2^)	0.7158	0.7771	0.7252
	C (mg/g)	9.9808	10.2976	11.6939
	*R*^*2*^	0.678	0.698	0.662
	Χ^2^	1.0678	1.0839	1.0370
	MPSD	6.2425	6.0824	5.3052

Literature reports that the adsorption process involves diffusion of the adsorbate towards the adsorbent surface that compete among themselves for adsorption onto the porous structure of the adsorbent^46^. With this consideration, the applicability of the Elovich model, besides the inter-particle diffusion model, was investigated. The Elovich model was also used to compute the initial adsorption rate, $\alpha$ with values highest for CWP (50.79 g/mg min) followed by AWP (24.10 g/mg min) and NWP (26.67 g/mg min) with non-significant values of *R*^2^ (0.771–0.803). The rate constant for inter-particle diffusion and the inter-particle diffusion model constant (mg/g) with sufficiently high values indicated the possibility of a boundary layer effect. Still, the corresponding very low values of *R*^2^ (0.662–0.698) discard the aptness to ascertain the mechanism for the adsorption^1^. The relatively high values of χ^2^ and MPSD for Elovich and the inter-particle diffusion model also limit their suitability for the studied systems.

### Effect of other metal ions on sequestration of Cr(VI)

The biosorbents were also used to sequester Cr(VI) in the presence of Na(I), Cu(II), Zn(II), As(III) and Co(III) metal ions (100 mg/L) from the binary metal ion solutions under the optimized conditions obtained from the earlier analysis. The presence of univalent, bivalent and trivalent metal ions had a negligible effect on the removal efficiency for Cr(VI). A similar effect has also been reported in other studies^39^. In a system with multiple metal ions, the active sites on the surface of the biosorbent are contested by these metal ions^47^. The effect of co-ions has been reported to depend upon the affinity of the adsorbent surface for the co-metal ions in the system. The biosorbent surface possibly has a high selectivity for Cr(VI) ions over the other ions in multi-metal systems^48^. Thus, the chemically modified biosorbents can efficiently sequester Cr(VI) even in multi-metal solutions.

### Mechanism for adsorption

The uptake of Cr(VI) onto biosorbents has been considered to involve various interactions depending upon the nature of the biosorbent^49^. The corresponding mechanism for the biosorption of Cr(VI) onto lignocellulosic biosorbent has been considered to involve:

1. Electrostatic interactions between the functional groups attached at the biosorbent surface and anionic forms of Cr(VI)^1^.
2. Ion exchange with the cations linked with the biosorbent surface^16^.
3. Reduction coupled adsorption of metal ion^50^.
4. Complexation of metal ions with the functionalities^4^.

**Figure 6:**
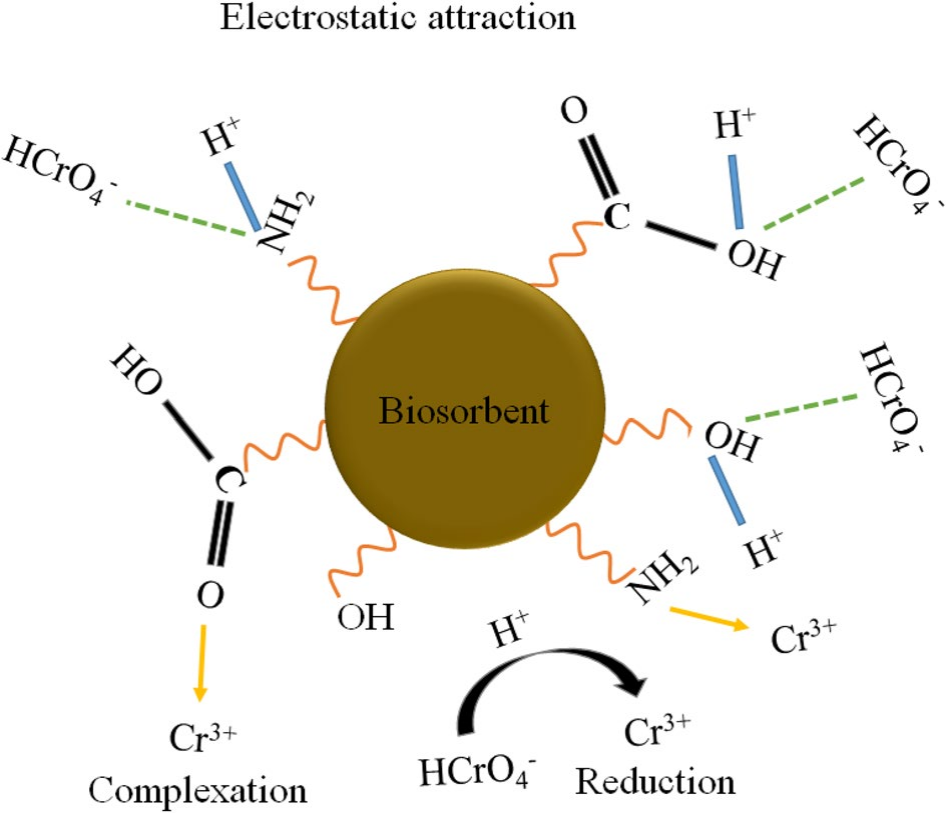
The mechanism for adsorption of Cr(VI) on biosorbents.

The plausible mechanism for biosorption of Cr(VI) onto lignocellulosic walnut shell derived biosorbents (NWP, AWP and CWP) can be justified based on the experimental observations (Fig. 6).

This report's analysis of pH studies specifies the role of plausible electrostatic interactions between Cr(VI) and the polyhydroxy, carboxy and amine functionalities present at the biosorbent surface^15^. The anionic form of Cr(VI) adsorbed on the positively charged surface of the biosorbents due to protonation at low pH. The thermodynamic studies confirm the endothermic nature of the adsorption, indicating the role of chemical interactions in the biosorption of Cr(VI) onto NWP, AWP and CWP. Ren et al. 2022 also reported Cr(VI) binding onto mycelial biomass by electrostatic interaction, redox reaction and complexation^5^. The on-course reduction of Cr(VI) in the highly acidic medium leads to generating Cr(III) ions. The shift in FTIR spectra peaks confirms the functionalities' participation at the surface of the biosorbents in the binding of Cr(III), possibly through complexation^16^. While in the case of AWP (modification with base), the ion exchange between the sodium ions linked with the polyhydroxy or carboxy functionalities and Cr(III) ions may also increase its adsorption capacity compared to NWP^5^. The enhancement of maximum adsorption capacity due to chemical treatment with citric acid leads to additional linking of carboxy functionalities onto the surface of CWP, resulting in better interactions with the cationic and anionic species in the solution^30^. Thus, treatment with citric acid enhances the efficiency of the waste walnut shell as a biosorbent for Cr(VI) from an aqueous medium.

## Conclusion

The biosorbents utilized in this study have been developed from walnut shell powder, a rich source of secondary metabolites containing polyhydroxy, carboxy and amine as the primary functional groups. The biosorbents NWP (derived from native walnut shell powder), AWP (chemical treatment with sodium hydroxide) and CWP (chemical treatment with citric acid) have been utilized for the sequestration of Cr(VI) from aqueous solution. The solution pH strongly impacted the uptake of Cr(VI) by the biosorbents. The adsorption data fitted well to Langmuir adsorption isotherm and followed pseudo-second-order kinetics. The maximum adsorption capacity of the biosorbents was obtained at pH 2 for CWP, followed by AWP and NWP. Citric acid enhanced the adsorption capacity of the biosorbent by modifying the active sites. Biosorption of Cr(VI) was found to be endothermic and spontaneous by chemical interaction between the anionic forms of Cr(VI) and the functionalities present at the biosorbent surface. Thus, the study confirms the beneficial role of citric acid in the surface modification of walnut shell powder. This solid waste can be used as an effective and eco-friendly biosorbent after treatment with citric acid to remove Cr(VI) from aqueous media.

## Supplementary Information

Supplementary Table S1.

## Data Availability

The datasets used and analyzed during the current study are available from the corresponding author upon reasonable request.
