# Supplementary material for: Rapid adsorptive removal of chromium from wastewater using walnut-derived biosorbents
Source: Sci Rep. 2023 Apr 26;13:6859. doi: 10.1038/s41598-023-33843-3 (PMC10133242; doi:10.1038/s41598-023-33843-3)
Supplement: Supplementary file 1 — Supplementary Table S1. [file 41598_2023_33843_MOESM1_ESM.doc]

Table S1. Thermodynamic parameters for the adsorption of Cr(VI) on biosorbents at different temperatures.

| Metal ion | T  (K) | ΔG^o^  (kJ/mol) | ΔH^o^  (kJ/mol) | ΔS^o^  (J/mol K) |
| --- | --- | --- | --- | --- |
| NWP | 288 | -47.13 | -22.36 | 86.01 |
|  | 298 | -25.63 |  |  |
|  | 308 | -26.49 |  |  |
|  | 318 | -27.35 |  |  |
|  | 328 | -28.21 |  |  |
| AWP | 288 | -44.05 | -20.44 | 81.96 |
|  | 298 | -44.87 |  |  |
|  | 308 | -45.69 |  |  |
|  | 318 | -46.51 |  |  |
|  | 328 | -47.33 |  |  |
| CWP | 288 | -38.22 | -16.96 | 73.84 |
|  | 298 | -38.96 |  |  |
|  | 308 | -39.70 |  |  |
|  | 318 | -40.44 |  |  |
|  | 328 | -41.18 |  |  |
